# Association between obstructive sleep apnea syndrome and blood pressure variability: a meta-analysis

**DOI:** 10.3389/fmed.2026.1882002

**Published:** 2026-07-06

**Authors:** Huijia Lei, Bo Kong, Yanwei Lv, Xiaozhu Ge

**Affiliations:** 1 Department of Otorhinolaryngology, Beijing Jishuitan Hospital, Capital Medical University, Beijing, China; 2 Department of Adult Cardiac Surgery, National Center for Cardiovascular Disease and Fuwai Hospital, Chinese Academy of Medical Sciences, Peking Union Medical College, Beijing, China; 3 Clinical Epidemiology research Center, Beijing Jishuitan Hospital, Capital Medical University, Beijing, China; 4 Department of Geriatrics, Beijing Jishuitan Hospital, Capital Medical University, Beijing, China

**Keywords:** blood pressure variability, hypertension, intermittent hypoxia, meta-analysis, obstructive sleep apnea syndrome

## Abstract

**Background:**

Obstructive sleep apnea syndrome (OSAS) is associated with increased cardiovascular risk, but its relationship with blood pressure variability (BPV), an independent predictor of adverse outcomes, remains incompletely defined. This meta-analysis aimed to evaluate the association between OSAS and BPV assessed by ambulatory blood pressure monitoring (ABPM).

**Methods:**

PubMed, Embase, Web of Science, Wanfang, and CNKI were systematically searched for observational studies comparing BPV between adults with OSAS and non-OSAS controls. BPV was evaluated based on standard deviation of systolic (SBP) and diastolic blood pressure (DBP) derived from 24-h ABPM, including nighttime, daytime, and 24-h periods, as reported in the original studies. Random-effects models were used to pool mean differences (MDs) with 95% confidence intervals (CIs).

**Results:**

Eleven studies comprising 17 datasets (1,261 patients with OSAS and 826 controls) were included. Compared with controls, OSAS was associated with significantly higher BPV across all periods. Nighttime BPV showed the largest differences (SBP: MD 1.89 mmHg, 95% CI 1.07–2.70; DBP: MD 1.93 mmHg, 95% CI 1.17–2.69), followed by 24-h BPV (SBP: MD 1.77 mmHg, 95% CI 0.80–2.75; DBP: MD 1.17 mmHg, 95% CI 0.47–1.87) and daytime BPV (SBP: MD 1.17 mmHg, 95% CI 0.35–1.99; DBP: MD 0.61 mmHg, 95% CI 0.26–0.96). Subgroup analyses suggested a trend toward increasing BPV with greater OSAS severity, with statistically significant differences observed only for nighttime DBP variability (*p* = 0.003). Meta-regression analyses showed that mean age, proportion of men, and mean body mass index were not significant modifiers of any BPV outcome (all *p* > 0.05). According to the GRADE framework, the certainty of evidence ranged from low to very low.

**Conclusion:**

OSAS is associated with increased BPV, particularly during nighttime. Although a trend toward greater BPV with increasing OSAS severity was observed, the certainty of evidence ranged from low to very low, and substantial between-study heterogeneity warrants cautious interpretation of the findings.

**Systematic review registration:**

CRD420261395221.

## Introduction

Obstructive sleep apnea syndrome (OSAS) is a common sleep-related breathing disorder characterized by recurrent episodes of upper airway collapse during sleep, leading to intermittent hypoxia, sleep fragmentation, and marked intrathoracic pressure swings (1, 2). Globally, OSAS affects approximately 9–38% of the adult population, with higher prevalence among men, older individuals, and those with obesity (3). In clinical practice, OSAS has been increasingly recognized as an important contributor to cardiovascular morbidity, being strongly associated with hypertension, coronary artery disease, heart failure, atrial fibrillation, and stroke (4, 5). Beyond its impact on mean blood pressure levels, OSAS may influence dynamic blood pressure regulation, thereby contributing to adverse cardiovascular outcomes (6, 7). Proposed mechanisms include sympathetic overactivation, endothelial dysfunction, oxidative stress, and inflammation, all of which may impair vascular compliance and autonomic control, ultimately promoting hemodynamic instability (6, 7).

Blood pressure variability (BPV) refers to fluctuations in blood pressure over time and can be broadly categorized into beat-to-beat (very short-term), 24-h (short-term), and visit-to-visit (long-term) variability (8, 9). Among these, short-term BPV derived from 24-h ambulatory blood pressure monitoring (ABPM) is most commonly used in clinical research, typically quantified by the standard deviation (SD) of systolic (SBP) and diastolic blood pressure (DBP) during daytime, nighttime, or across the full 24-h period (9–11). Increasing evidence suggests that elevated BPV is independently associated with target organ damage and adverse cardiovascular outcomes, potentially through mechanisms involving vascular shear stress, arterial stiffness, and impaired baroreflex sensitivity. Several pathophysiological pathways may link OSAS to increased BPV (12–14). Recurrent nocturnal hypoxia and arousals can trigger sustained sympathetic activation and altered circadian blood pressure regulation, while sleep fragmentation and endothelial dysfunction may further destabilize vascular tone (7, 15). Several clinical studies have reported an association between OSAS and increased BPV. For example, Shi et al. demonstrated that severe OSAS was associated with significantly increased systolic and diastolic BPV in hypertensive patients (16), while Ke et al. reported higher 24-h systolic BPV among patients with OSAS and suggested a link with cardiovascular risk (17). However, the available studies have varied considerably in sample size, patient characteristics, BPV assessment periods (nighttime, daytime, or 24-h), and OSAS severity, resulting in inconsistent estimates regarding the magnitude and clinical relevance of the association (18, 19). To date, no meta-analysis has systematically summarized the relationship between OSAS and BPV. Therefore, this study was conducted to quantitatively synthesize available evidence comparing BPV between adults with OSAS and non-OSAS controls and to explore whether the association varies according to the severity of OSAS.

## Methods

The meta-analysis was carried out in accordance with established methodological guidance, following the principles outlined in the PRISMA 2020 statement (20) and the Cochrane Handbook for Systematic Reviews of Interventions (21), encompassing protocol planning, study selection, data collection, statistical analysis, and results interpretation. The study protocol was registered prospectively in the PROSPERO database (registration number: CRD420261395221).

### Database search

A systematic literature search was conducted in PubMed, Embase, Web of Science, Wanfang, and China National Knowledge Infrastructure (CNKI) to identify studies that met the eligibility criteria for inclusion. The search strategy was constructed using the combination of the following terms: (1) “obstructive sleep apnea” OR “OSA” OR “OSAS” OR “sleep-disordered breathing”; (2) “blood pressure variability” OR “BP variability” OR “BPV” OR “blood pressure variation” OR “blood pressure fluctuation” OR “short-term blood pressure variability” OR “24-h blood pressure variability” OR “ambulatory blood pressure variability” OR “standard deviation of blood pressure”; and (3) “ABPM” OR “ambulatory blood pressure monitoring” OR “24-h blood pressure” OR “24 h blood pressure” OR “daytime blood pressure” OR “nighttime blood pressure” OR “nocturnal blood pressure.” Only full-text articles published in English or Chinese in peer-reviewed journals and involving human participants were eligible for inclusion. Additionally, the reference lists of relevant reviews and original studies were manually examined to identify further potentially eligible publications. All databases were searched from their inception up to March 25, 2026. Detailed search strategies for each database are presented in Supplementary File 1.

### Study inclusion and exclusion criteria

The selection of studies was guided by the PICOS principle:

- Population (P): Adult participants (≥18 years), including general or specific populations (e.g., hypertensive patients).
- Intervention/Exposure (I): OSAS diagnosed using objective methods such as polysomnography (PSG) or validated portable monitoring devices, typically defined by an apnea–hypopnea index (AHI) ≥ 5 events/h.
- Comparison (C): Individuals without OSAS.
- Outcomes (O): BPV assessed as the SD of SBP or DBP, derived from 24-h ABPM, including nighttime, daytime, and 24-h measurements.
- Study design (S): Observational studies, including case–control, cross-sectional, and cohort studies, reporting comparative data between OSAS and non-OSAS groups.

Studies were excluded if they (1) included pediatric populations (<18 years); (2) did not include a non-OSAS comparison group or lacked clear exposure classification; (3) defined OSAS without objective diagnostic methods (e.g., self-reported only); (4) did not assess BPV using ABPM-derived SD of SBP or DBP (e.g., studies reporting only dipping status, blood pressure levels, or non-standard BPV metrics); (5) did not provide sufficient data for extraction or calculation of effect estimates; or (6) were reviews, editorials, case reports, conference abstracts without adequate data, or animal studies. For studies with overlapping participants, those with the largest sample size were included in the meta-analysis.

### Study quality assessment

Two reviewers independently performed the literature search, screened studies for eligibility, extracted data, and assessed study quality. Any disagreements were resolved through discussion, and when necessary, a third investigator was consulted to reach consensus. The methodological quality of the included studies was evaluated using the Newcastle–Ottawa Scale (NOS) (22), which assesses study quality across three domains: selection, comparability, and outcome assessment. NOS scores range from 1 to 9, with studies scoring ≥7 considered to be of high quality.

### Data collection

Data extraction was conducted independently by two reviewers using a standardized data collection form. Extracted variables included study characteristics (first author, publication year, country, and study design), participant characteristics (diagnostic criteria for OSAS, number of patients with OSAS, mean AHI for patients with OSAS, source and number of controls, mean age, sex distribution, mean body mass index [BMI], hypertensive status of the participants, and mean SBP and DBP levels), ABPM protocol, metrics for BPV (SD of nighttime, daytime, or 24-h SBP/DBP), and variables matched or adjusted between participants with and without OSAS when BPV metrics were compared.

### Statistical analyses

We separately compared the SDs of nighttime, daytime, and 24-h SBP and DBP between patients with OSAS and controls as the indicators of BPV, which were summarized as mean differences (MDs) and their corresponding 95% confidence intervals (CIs) (21). Studies reporting multiple OSAS severity categories (e.g., mild, moderate, and severe) were included by treating each severity level as an independent comparison against the shared control group, with the control sample size appropriately divided to avoid double counting (21). To evaluate heterogeneity across studies, we applied the Cochrane Q test and calculated the *I*^2^ statistic (23). Heterogeneity was categorized based on *I*^2^ values as low (<25%), moderate (25–75%), or high (>75%). Pooled effect estimates were calculated using the inverse variance (IV) approach within a random-effects framework (DerSimonian–Laird method) to account for potential between-study variability (21). In addition, 95% prediction intervals (PIs) were calculated for each pooled analysis to estimate the range within which the true effect of a future study is expected to lie, taking into account both within-study and between-study heterogeneity (21). Sensitivity analyses were performed using a leave-one-out approach, in which each study was sequentially excluded to assess the stability and robustness of the pooled results (24). Predefined subgroup analyses were performed according to OSAS severity, categorized by AHI as mild OSAS (5–14 events/h), moderate OSAS (15–30 events/h), and severe OSAS (>30 events/h). To further explore potential sources of heterogeneity, univariate random-effects meta-regression analyses were performed using study-level demographic characteristics that were consistently reported across the included studies, including mean age, proportion of men, and mean BMI (21). Publication bias was assessed through visual inspection of funnel plot symmetry and further evaluated using Egger’s regression test (25). A two-sided *p* value < 0.05 was considered indicative of statistical significance. All analyses were performed using RevMan (version 5.3; Cochrane Collaboration, Oxford, UK) and Stata (version 17.0; StataCorp, College Station, TX, USA). Certainty of evidence for each outcome was evaluated using the Grading of Recommendations Assessment, Development and Evaluation (GRADE) framework (21). Because all included studies were observational and predominantly cross-sectional, the certainty of evidence started at low. The certainty was further downgraded when important limitations were identified, including substantial unexplained heterogeneity. The final certainty of evidence was categorized as high, moderate, low, or very low.

## Results

### Database search results

**Figure 1:**
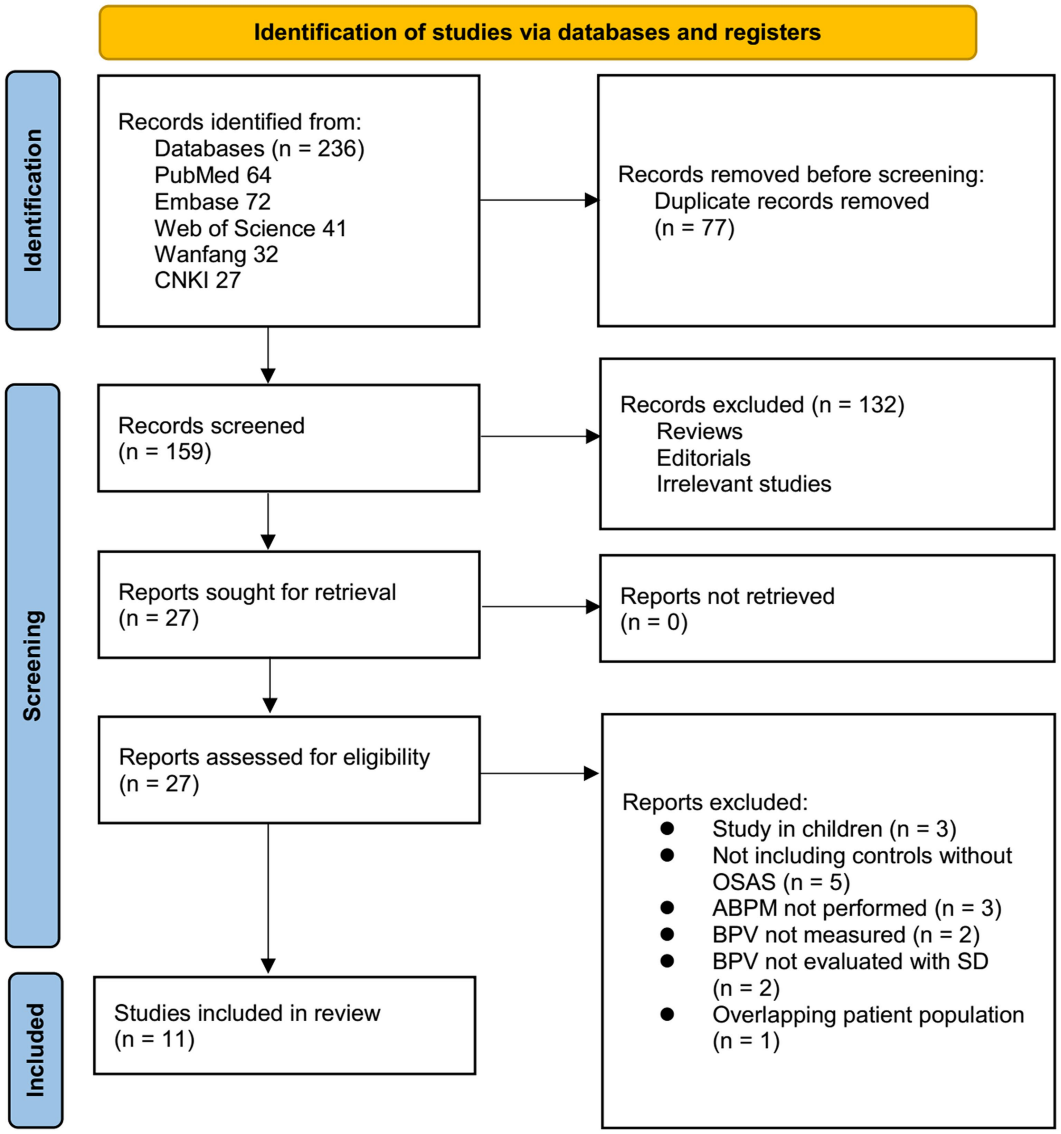
Flow diagram of the study selection process.

The study selection process is illustrated in Figure 1. A total of 236 records were retrieved from the 5 databases, of which 77 duplicates were removed. Following title and abstract screening, 132 records were excluded for not meeting the predefined inclusion criteria. The full texts of 27 articles were subsequently evaluated independently by 2 reviewers, and 16 were excluded for the reasons detailed in Figure 1. Ultimately, 11 studies met the eligibility criteria and were included in the quantitative meta-analysis (16, 17, 26–34).

### Overview of study characteristics

The main characteristics of the included studies are summarized in Table 1. A total of 11 studies (16, 17, 26–34) were included, yielding 17 independent datasets, as three studies (26, 29, 33) reported results stratified by OSAS severity (mild, moderate, and severe), which were treated as separate comparisons. All included studies adopted a cross-sectional design and were conducted between 2011 and 2025, predominantly in China (16, 17, 26, 29–34), with additional studies from Japan (28) and Brazil (27), indicating limited geographic diversity. The sample sizes varied substantially across studies, with the number of OSAS participants ranging from 25 to 212, and control groups from 11 to 222, reflecting variability in study scale. Overall, 1,261 patients with OSAS and 826 controls without OSAS were included. Ten of the studies included hypertensive populations (16, 17, 26–31, 33, 34), while another study included both hypertensive and non-hypertensive patients (32). OSAS was diagnosed using PSG (17, 26, 28–34) or portable PSG devices (16, 27) with AHI-based definitions. The mean age of participants ranged from approximately 43–71 years, and the proportion of men ranged from 50.2 to 100%, suggesting a predominantly middle-aged to elderly population with a male predominance in several studies. BPV was assessed using 24-h ABPM, with relatively consistent protocols involving measurements at 15–30-min intervals during daytime and 20–60-min intervals during nighttime. All studies defined BPV using the SD of SBP and DBP, reported across daytime, nighttime, and/or 24-h periods. All the included studies controlled for important confounding factors, typically including age, sex, and BMI, with additional consideration of clinical variables such as cardiovascular disease, metabolic parameters, and medication use in some studies to a varying degree (16, 17, 28–32, 34). Four studies (17, 28, 31, 32) additionally adjusted for antihypertensive medication use, as summarized in Table 1, although detailed information regarding medication classes and treatment regimens was not consistently available.

**Table 1:** Characteristics of the included studies.

Study	Country	Design	Methods for the diagnosis of OSAS	No. of subjects with OSAS	Mean AHI in OSAS	Source of controls	No. of controls	Mean age (years)	Men (%)	Mean BMI (kg/m2)	HTN (%)	Mean SBP (mmHg)	Mean DBP (mmHg)	ABPM protocol	Metric for BPV	Variables matched or adjusted
Wang 2011 (26) mild	China (Urumqi)	CS	Full PSG, AHI 5–14	35	10.2	Hypertensive patients without OSAS from same hospital	12	43.6	83.3	29.6	100	NR	NR	Daytime 08:00–23:00 (every 20 min); Nighttime 23:00–08:00 (every 20 min) for 24 h	SD for nighttime, daytime SBP and DBP	Age, sex, and BMI
Wang 2011 (26) moderate	China (Urumqi)	CS	Full PSG, AHI 15–30	25	28.3	Hypertensive patients without OSAS from same hospital	11	43.4	86	28.6	100	NR	NR	Daytime 08:00–23:00 (every 20 min); Nighttime 23:00–08:00 (every 20 min) for 24 h	SD for nighttime, daytime SBP and DBP	Age, sex, and BMI
Wang 2011 (26) severe	China (Urumqi)	CS	Full PSG, AHI > 30	32	59	Hypertensive patients without OSAS from same hospital	11	44.4	85.8	29.2	100	NR	NR	Daytime 08:00–23:00 (every 20 min); Nighttime 23:00–08:00 (every 20 min) for 24 h	SD for nighttime, daytime SBP and DBP	Age, sex, and BMI
Steinhorst 2014 (27)	Brazil	CS	Portable PSG, AHI > 10 for severe OSA	57	23.1	Hypertensive patients without OSAS from same clinic	50	58.5	50.2	29.1	100	135.6	81.8	Daytime 07:00–23:00 (every 15 min); Nighttime 23:00–07:00 (every 20 min), for 24 h	SD for nighttime, daytime, and 24 h SBP and DBP	Age, sex, BMI, and respective BP
Sasaki 2015 (28)	Japan	CS	Full PSG, AHI ≥ 15 or AHI ≥ 5 with symptoms	212	32.9	Hypertensive patients without OSAS from same hospital	82	61.1	90.5	25.8	100	133.8	85.9	Every 30 min throughout 24 h	SD for nighttime and daytime SBP and DBP	Age, sex, BMI, sleep BP, AHI, arousal index, lowest SpO₂, REM%, PLMI, CVD, and antihypertensive medications
Shi 2017 (16)	China (Harbin)	CS	Portable PSG, AHI > 30 for severe OSA	43	59.6	Hypertensive patients without OSA from same hospital	43	47.3	75.6	27.4	100	136.8	86.9	Daytime 06:00–22:00 (every 20 min); Nighttime 22:00–06:00 (every 30 min) for 24 h	SD for nighttime, daytime, and 24 h SBP and DBP	Age, sex, BMI, neck circumference, HR, and snoring history
Ke 2017 (17)	China (Guangzhou)	CS	Full PSG, AHI ≥ 5	162	11.6	Hypertensive patients without OSA from same cohort	222	49.5	64.1	25.2	100	130.5	77.3	Minimum 20 awake and 7 asleep valid readings, for 24 h	SD for nighttime, daytime, and 24 h SBP and DBP	Age, sex, BMI, smoking, Hs-CRP, CVD, and antihypertensive medications
Wang 2017 (29) mild	China (Hengshui)	CS	Full PSG, AHI 5–14	46	10.9	Hypertensive patients without OSA from same hospital	13	82.7	64.4	25.6	100	139.2	64	Daytime 06:00–22:00 (every 30 min); Nighttime 22:00–06:00 (every 60 min) for 24 h	SD for nighttime, daytime, and 24 h SBP and DBP	Age, sex, BMI, smoking history, blood lipids, and SCr
Wang 2017 (29) moderate	China (Hengshui)	CS	Full PSG, AHI 15–30	48	23.4	Hypertensive patients without OSA from same hospital	13	84.2	63.9	25.8	100	141	65.8	Daytime 06:00–22:00 (every 30 min); Nighttime 22:00–06:00 (every 60 min) for 24 h	SD for nighttime, daytime, and 24 h SBP and DBP	Age, sex, BMI, smoking history, blood lipids, and SCr
Wang 2017 (29) severe	China (Hengshui)	CS	Full PSG, AHI > 30	40	53.1	Hypertensive patients without OSA from same hospital	12	83.5	67.3	25.9	100	145.3	69.7	Daytime 06:00–22:00 (every 30 min); Nighttime 22:00–06:00 (every 60 min) for 24 h	SD for nighttime, daytime, and 24 h SBP and DBP	Age, sex, BMI, smoking history, blood lipids, and SCr
Shao 2018 (30)	China (Urumqi)	CS	Full PSG, AHI ≥ 15	41	NR	Hypertensive males without OSA from same center	52	44.3	100	27.9	100	131.5	88.2	Daytime 08:00–22:00 (every 15 min); Nighttime 22:00–08:00 (every 20 min), for 24 h	SD for nighttime, daytime, and 24 h SBP and DBP	Age, sex, BMI, and TST
Zhang 2023 (32)	China (Beijing)	CS	Full PSG, AHI ≥ 15	72	NR	Patients without OSA from same center	72	71.5	74.5	26.7	78.5	138.4	77.5	Minimum 20 awake and 7 asleep valid readings, for 24 h	SD for nighttime, daytime, and 24 h SBP and DBP	Age, sex, BMI, mean BP, HR, smoking history, amd CV medications
Fei 2023 (31)	China (Changsha)	CS	Full PSG, AHI ≥ 5	114	NR	Hypertensive patients without OSA from same center	114	62	73.7	24.2	100	135.5	70	Daytime 06:00–22:00 (every 30 min); Nighttime 22:00–06:00 (every 60 min) for 24 h	SD for nighttime, daytime, and 24 h SBP and DBP	Age, sex, BMI, FBG, blood lipids, SCr, duration of HTN, and CV medications
Xu 2024 (33) mild	China (Heifei)	CS	Full PSG, AHI 5–14	74	NR	Hypertensive patients without OSA from same hospital	13	62.1	60.1	25.6	100	NR	NR	Daytime (every 30 min); Nighttime (every 60 min) for 24 h	SD for nighttime, daytime, and 24 h SBP and DBP	Age, sex, and BMI
Xu 2024 (33) moderate	China (Heifei)	CS	Full PSG, AHI 15–30	68	NR	Hypertensive patients without OSA from same hospital	13	61.4	77.5	26.3	100	NR	NR	Daytime (every 30 min); Nighttime (every 60 min) for 24 h	SD for nighttime, daytime, and 24 h SBP and DBP	Age, sex, and BMI
Xu 2024 (33) severe	China (Heifei)	CS	Full PSG, AHI > 30	83	NR	Hypertensive patients without OSA from same hospital	14	56	79.8	28.5	100	NR	NR	Daytime (every 30 min); Nighttime (every 60 min) for 24 h	SD for nighttime, daytime, and 24 h SBP and DBP	Age, sex, and BMI
Qiu 2025 (34)	China (Shenzhen)	CS	Full PSG, AHI ≥ 5	109	30.1	Hypertensive patients without OSA from same hospital	79	57.9	50.4	25.8	100	123.7	79.2	Daytime 08:00–22:00 (every 20 min); Nighttime 22:00–08:00 (every 30 min), for 24 h	SD for nighttime, daytime, and 24 h SBP and DBP	Age, sex, BMI, comorbidities, and smoking history

AHI, apnea–hypopnea index; OSAS, obstructive sleep apnea syndrome; CS, cross-sectional; PSG, polysomnography; BMI, body mass index; HTN, hypertension; SBP, systolic blood pressure; DBP, diastolic blood pressure; ABPM, ambulatory blood pressure monitoring; BPV, blood pressure variability; SD, standard deviation; REM, rapid eye movement; SpO_2_, peripheral oxygen saturation; PLMI, periodic limb movement index; CVD, cardiovascular disease; HR, heart rate; Hs-CRP, high-sensitivity C-reactive protein; SCr, serum creatinine; TST, total sleep time; FBG, fasting blood glucose; NR, not reported.

### Study quality evaluation

The methodological quality of the included studies was assessed using the NOS, with detailed results presented in Table 2. The NOS scores ranged from 7 to 9, indicating high overall study quality. A total of six datasets from three studies (17, 31, 33) achieved the maximum score of 9, reflecting strong methodological rigor, including appropriate case definition, adequate control selection, and comprehensive adjustment for confounding factors. Nine datasets from five studies (16, 26, 28–30) scored 8, suggesting generally good quality with minor limitations, most commonly related to representativeness of cases or lack of information on non-response rates. Three studies scored 7 (27, 32, 34), primarily due to limited representativeness or incomplete control of confounding variables. Overall, case definition, exposure ascertainment, and comparability between groups were generally adequate across studies, and all analyses controlled for key confounders such as age, sex, and BMI. However, some variability existed in the extent of adjustment for additional clinical factors and in the representativeness of study populations. Taken together, the included studies were considered to be of moderate to high quality, supporting the reliability of the pooled results while acknowledging potential heterogeneity arising from study design and population characteristics.

**Table 2:** Study quality evaluation via the Newcastle-Ottawa Scale.

Study	Adequate definition of the cases	Representativeness of the cases	Selection of controls	Definition of controls	Controlled for age and sex	Controlled for other factors	Ascertainment of exposure	Same method of ascertainment for cases and controls	Non-response rate	Overall
Wang 2011 (26) mild	1	1	1	1	1	1	1	1	0	8
Wang 2011 (26) moderate	1	1	1	1	1	1	1	1	0	8
Wang 2011 (26) severe	1	1	1	1	1	1	1	1	0	8
Steinhorst 2014 (27)	1	0	1	1	1	1	1	1	0	7
Sasaki 2015 (28)	1	1	1	1	1	1	1	0	1	8
Shi 2017 (16)	1	1	1	1	1	1	1	1	0	8
Ke 2017 (17)	1	1	1	1	1	1	1	1	1	9
Wang 2017 (29) mild	1	0	1	1	1	1	1	1	1	8
Wang 2017 (29) moderate	1	0	1	1	1	1	1	1	1	8
Wang 2017 (29) severe	1	0	1	1	1	1	1	1	1	8
Shao 2018 (30)	1	1	1	1	1	1	1	1	0	8
Zhang 2023 (32)	1	0	1	1	1	1	1	1	0	7
Fei 2023 (31)	1	1	1	1	1	1	1	1	1	9
Xu 2024 (33) mild	1	1	1	1	1	1	1	1	1	9
Xu 2024 (33) moderate	1	1	1	1	1	1	1	1	1	9
Xu 2024 (33) severe	1	1	1	1	1	1	1	1	1	9
Qiu 2025 (34)	1	0	1	1	1	1	1	1	0	7

### Influence of OSAS on nighttime BPV

**Figure 2:**
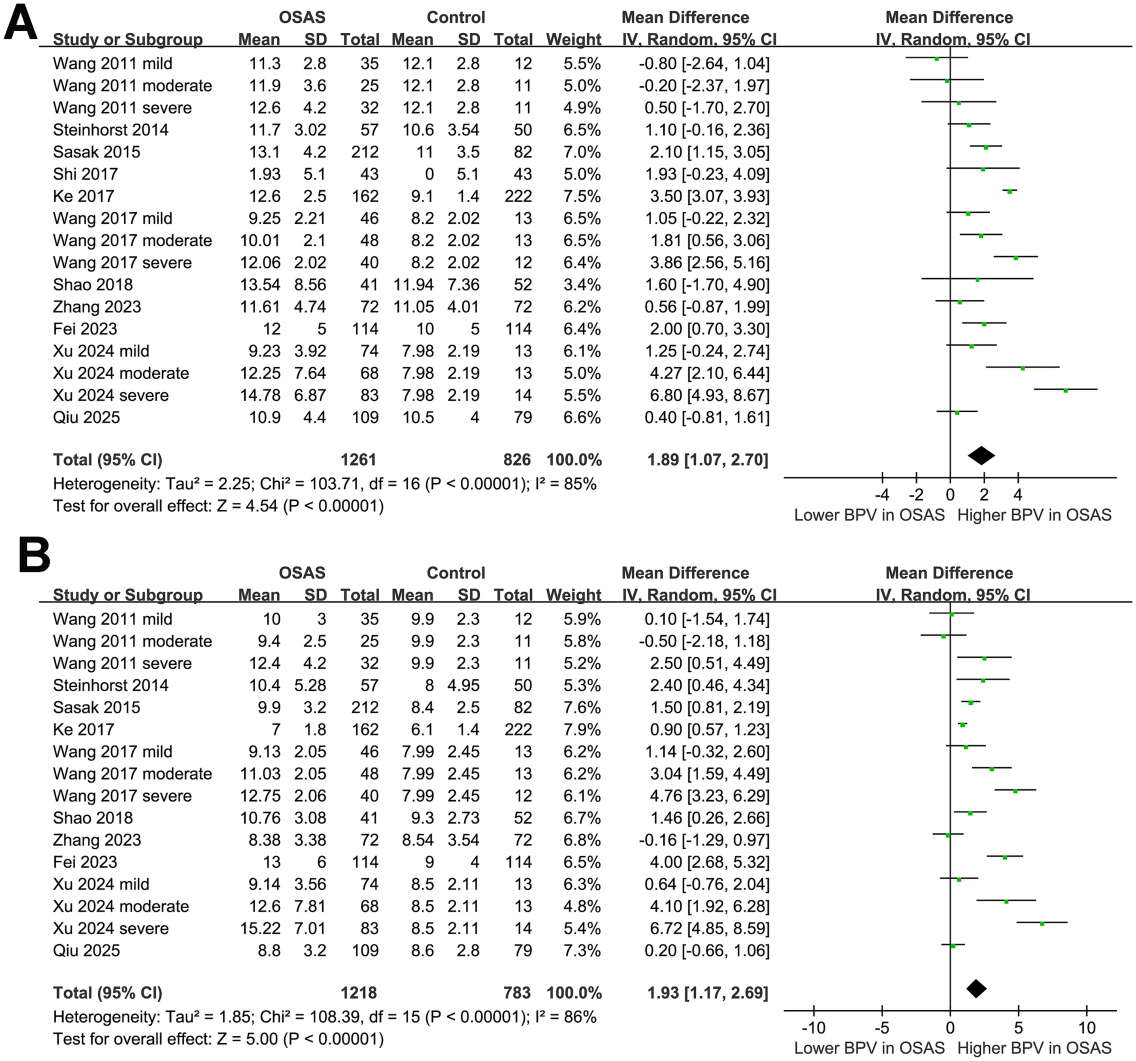
Forest plots showing the meta-analysis comparing nighttime BPV as evaluated by SD of SBP and DBP between patients with OSAS and controls: **(A)** forest plots for the meta-analysis comparing SD of nighttime SBP; and **(B)** forest plots for the meta-analysis comparing SD of nighttime DBP.

The pooled analysis of the 17 datasets from 11 studies (16, 17, 26–34) showed that OSAS was associated with an increased SD of nighttime SBP compared to controls (MD: 1.89 mmHg, 95% CI: 1.07–2.70, *p* < 0.001; *I*^2^ = 85%; Figure 2A). The corresponding 95% PI ranged from −1.43 to 5.21 mmHg, indicating substantial between-study variability in the magnitude of the association. Leave-one-out sensitivity analyses showed similar results, with MD ranging from 1.61 to 2.04 mmHg (all *p* < 0.05), indicating the robustness of the overall estimate. Further subgroup analyses by OSAS severity suggested a progressive increase in nighttime SBP variability, with MDs of 0.64, 1.95, and 3.34 mmHg for mild, moderate, and severe OSAS compared with controls, respectively. However, there was no clear evidence of a difference between subgroups (*p* for subgroup difference = 0.12; Supplementary Figure S1A). Similarly, the meta-analysis of 16 datasets from 10 studies (17, 26–34) also suggested an increased SD of nighttime DBP in OSAS compared with controls (MD: 1.93 mmHg, 95% CI: 1.17–2.69, *p* < 0.001; *I*^2^ = 86%; Figure 2B). The corresponding 95% PI ranged from −1.10 to 4.96 mmHg. Sensitivity analyses by omitting one dataset at a time showed consistent results (MD: 1.63–2.08 mmHg, all *p* < 0.05). Further subgroup analyses suggested a trend toward greater nighttime DBP variability with increasing OSAS severity (MD: 0.67, 2.17, and 4.68 mmHg for mild, moderate, and severe OSAS vs. controls, respectively), with evidence of a difference between subgroups (*p* for subgroup difference = 0.003; Supplementary Figure S1B).

### Influence of OSAS on daytime BPV

**Figure 3:**
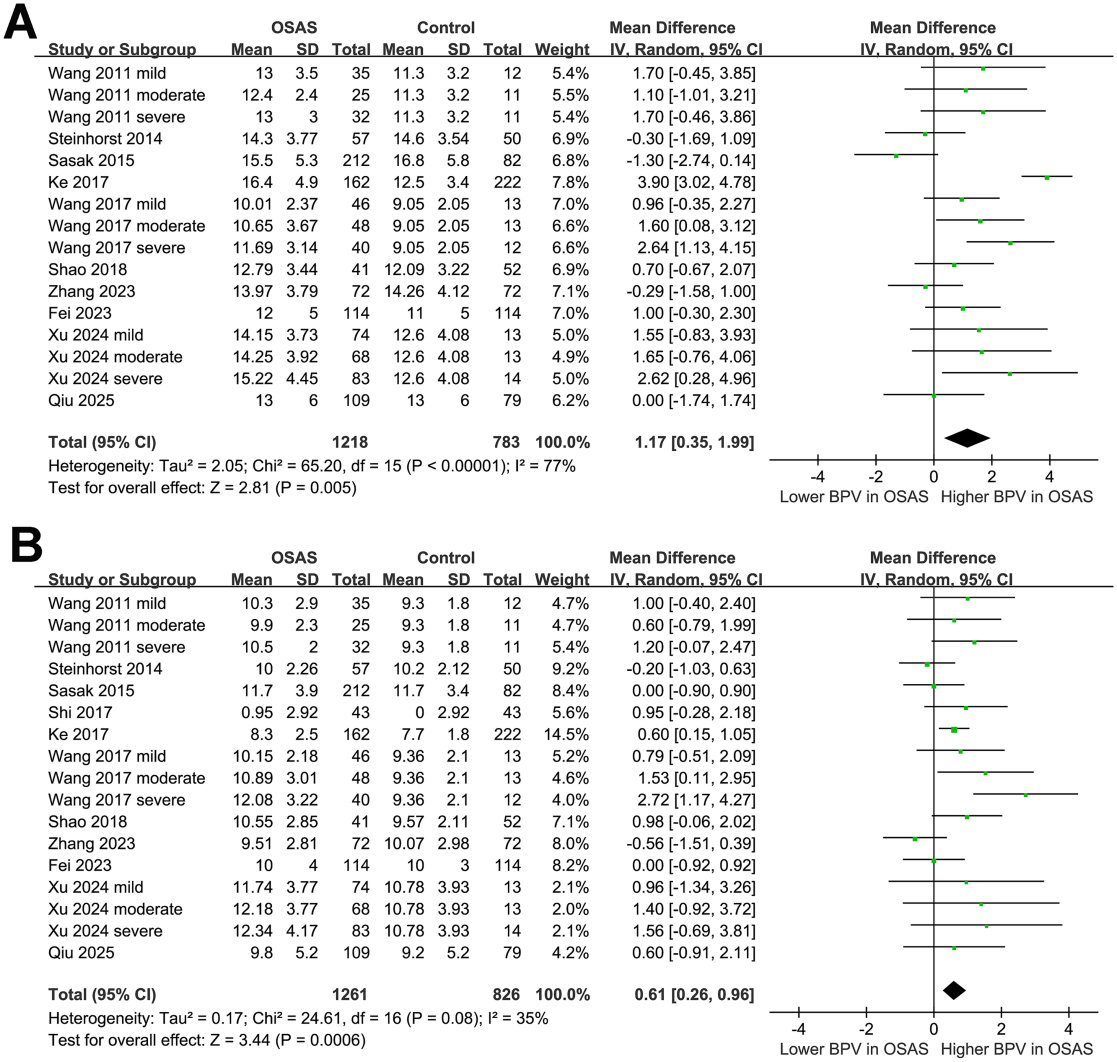
Forest plots showing the meta-analysis comparing daytime BPV as evaluated by SD of SBP and DBP between patients with OSAS and controls: **(A)** forest plots for the meta-analysis comparing SD of daytime SBP; and **(B)** forest plots for the meta-analysis comparing SD of daytime DBP.

Further meta-analyses with 16 datasets from 10 studies (17, 26–34) and 17 datasets from 11 studies (16, 17, 26–34) showed that patients with OSAS had higher daytime BP variability, as reflected by increased SDs of SBP (MD: 1.17 mmHg, 95% CI: 0.35 to 1.99, *p* = 0.005; *I*^2^ = 77%; Figure 3A) and DBP (MD: 0.61 mmHg, 95% CI: 0.26 to 0.96, *p* < 0.001; *I*^2^ = 35%; Figure 3B). The corresponding 95% PIs ranged from −2.03 to 4.37 mmHg for the SD of SBP and −0.35 to 1.57 mmHg for the SD of DBP. Sensitivity analyses, performed by sequentially excluding individual datasets, yielded consistent results, with pooled MDs ranging from 0.87 to 1.35 mmHg for SBP and from 0.49 to 0.69 mmHg for DBP (all *p* < 0.05). Subgroup analyses further suggested a numerical increase in daytime SBP and DBP variability with increasing OSAS severity (SD-SBP: MD 1.23, 1.48, and 2.39 mmHg; SD-DBP: MD 0.90, 1.11, and 1.50 mmHg for mild, moderate, and severe OSAS vs. controls, respectively), although the subgroup differences are not statistically significant (*p* = 0.28 and 0.59, respectively; Supplementary Figures S2A,B).

### Influence of OSAS on 24-h BPV

**Figure 4:**
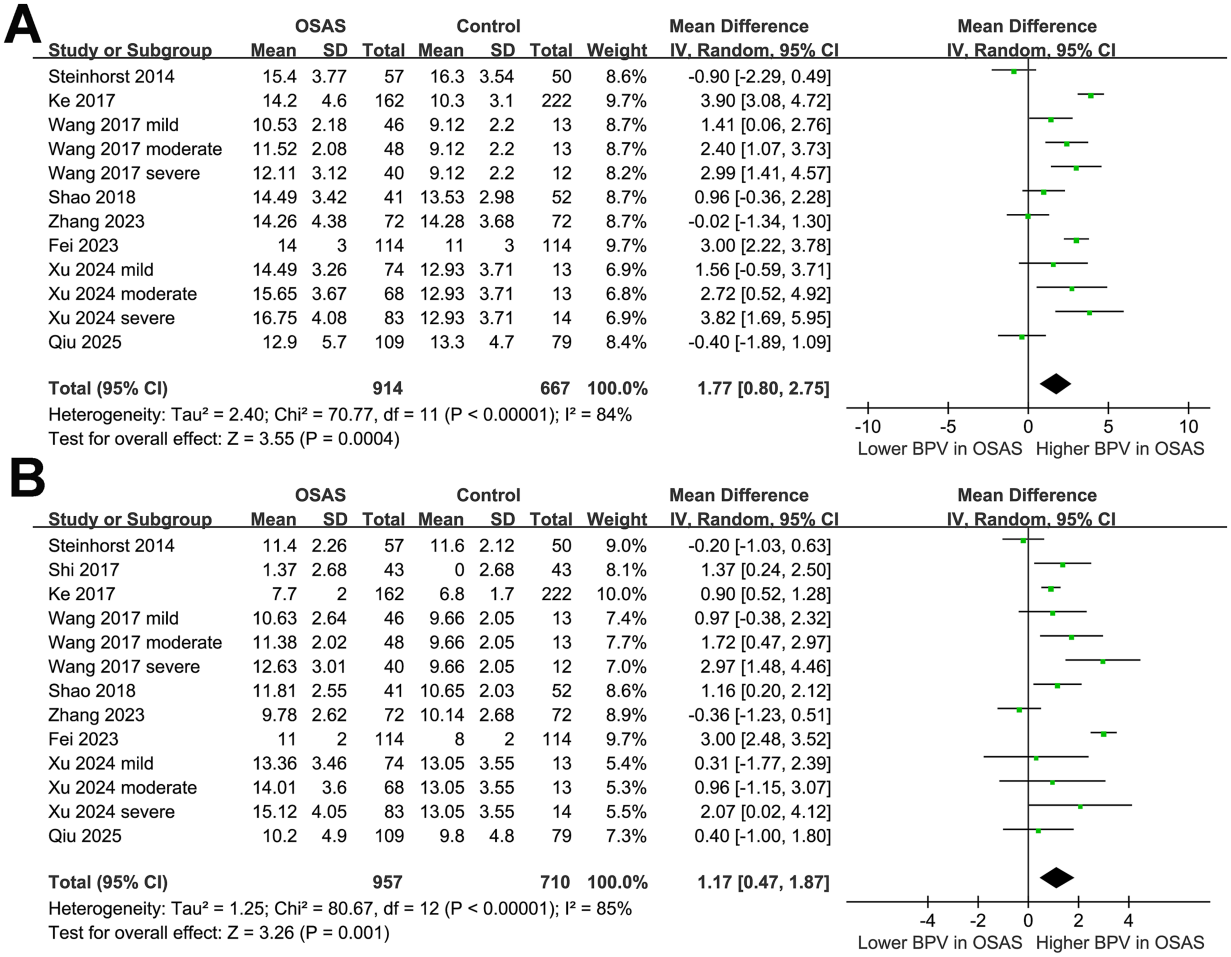
Forest plots showing the meta-analysis comparing 24-h BPV as evaluated by SD of SBP and DBP between patients with OSAS and controls: **(A)** forest plots for the meta-analysis comparing SD of 24-h SBP; and **(B)** forest plots for the meta-analysis comparing SD of 24-h DBP.

Finally, the pooled analyses with 12 datasets from eight studies (17, 27, 29–34) and 13 datasets from nine studies (16, 17, 27, 29–34) showed that patients with OSAS also had a higher 24-h BPV as compared to controls, as evaluated by increased SDs of 24-h SBP (MD: 1.77 mmHg, 95% CI: 0.80–2.75, *p* < 0.001; *I*^2^ = 84%; Figure 4A) and DBP (MD: 1.17 mmHg, 95% CI: 0.47–1.87, *p* = 0.001; *I*^2^ = 85%; Figure 4B). The corresponding 95% PIs for SDs of SBP and DBP ranged from −1.86 to 5.40 mmHg and −1.41 to 3.75 mmHg, respectively. Sensitivity analyses by sequentially excluding individual datasets retrieved consistent results, with pooled MDs ranging from 1.54 to 2.03 mmHg for SBP and from 0.91 to 1.32 mmHg for DBP (all *p* < 0.05). Subgroup analyses suggested a trend toward increasing 24-h SBP and DBP variability with greater OSAS severity (SD-SBP: MD 1.45, 2.49, and 3.28 mmHg; SD-DBP: MD 0.77, 1.52, and 2.04 mmHg for mild, moderate, and severe OSAS vs. controls, respectively), although there was no clear evidence of differences between subgroups (*p* for subgroup difference = 0.11 and 0.27, respectively; Supplementary Figures S3A,B).

### Meta-regression analyses

To investigate potential sources of heterogeneity, univariate meta-regression analyses were performed using mean age, proportion of men, and mean BMI as covariates (Table 3). None of these variables significantly modified the association between OSAS and nighttime, daytime, or 24-h BPV as evaluated by SDs of SBP or DBP (all *p* > 0.05). These findings suggest that the observed between-study heterogeneity was not substantially explained by differences in demographic characteristics across the included studies.

**Table 3:** Meta-regression analyses for potential sources of heterogeneity.

Outcome	Covariate	Datasets (*n*)	Coefficient	95% CI	*p* value
Nighttime SBP variability	Mean age (years)	17	0.022	−0.038–0.083	0.468
	Men (%)	17	0.006	−0.057–0.070	0.841
	Mean BMI (kg/m^2^)	17	−0.245	−0.760–0.270	0.351
Nighttime DBP variability	Mean age (years)	16	0.040	−0.016–0.096	0.159
	Men (%)	16	0.005	−0.049–0.060	0.849
	Mean BMI (kg/m^2^)	16	−0.072	−0.546–0.402	0.766
Daytime SBP variability	Mean age (years)	16	−0.007	−0.066–0.053	0.829
	Men (%)	16	−0.008	−0.068–0.051	0.778
	Mean BMI (kg/m^2^)	16	−0.075	−0.582–0.433	0.773
Daytime DBP variability	Mean age (years)	17	0.004	−0.023–0.031	0.782
	Men (%)	17	0.006	−0.019–0.031	0.638
	Mean BMI (kg/m^2^)	17	0.029	−0.183–0.241	0.789
24-h SBP variability	Mean age (years)	12	0.003	−0.072–0.079	0.932
	Men (%)	12	0.035	−0.039–0.110	0.353
	Mean BMI (kg/m^2^)	12	−0.482	−1.187–0.224	0.181
24-h DBP variability	Mean age (years)	13	0.015	−0.039–0.068	0.590
	Men (%)	13	0.025	−0.029–0.079	0.373
	Mean BMI (kg/m^2^)	13	−0.318	−0.821–0.185	0.215

BMI, body mass index; CI, confidence interval; DBP, diastolic blood pressure; OSAS, obstructive sleep apnea syndrome; SBP, systolic blood pressure.

### Publication bias

**Figure 5:**
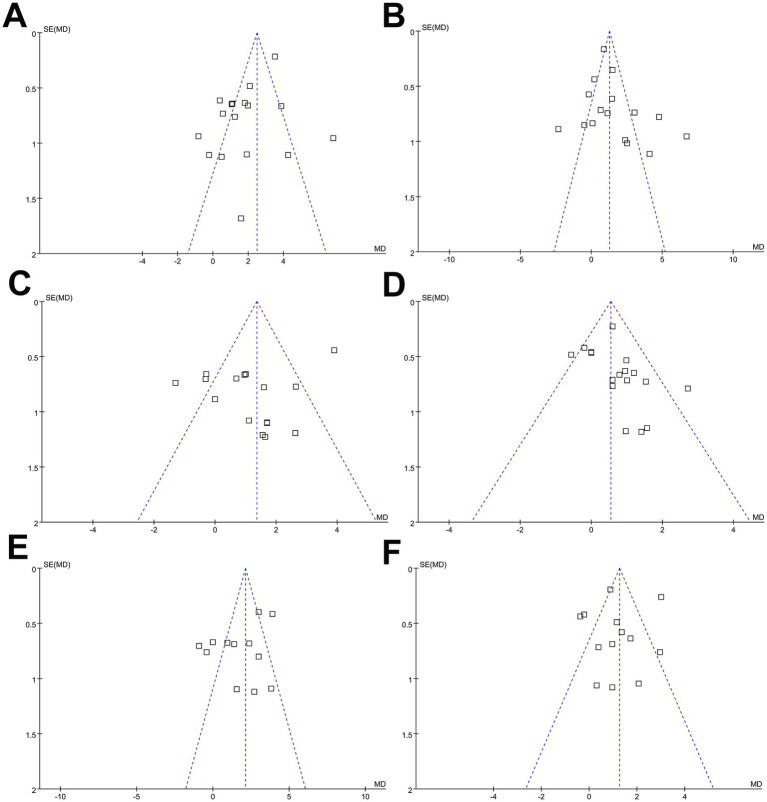
Funnel plots evaluating the publication bias for the meta-analyses: **(A)** funnel plots for the meta-analysis comparing SD of nighttime SBP; **(B)** funnel plots for the meta-analysis comparing SD of nighttime DBP; **(C)** funnel plots for the meta-analysis comparing SD of daytime SBP; **(D)** funnel plots for the meta-analysis comparing SD of daytime DBP; **(E)** funnel plots for the meta-analysis comparing SD of 24-h SBP; and **(F)** funnel plots for the meta-analysis comparing SD of 24-h DBP.

As illustrated in Figures 5A–F, the funnel plots assessing the meta-analyses comparing the BPV of nighttime, daytime, and 24-h SBP and DBP between patients with OSAS and controls appeared generally symmetrical. In line with this observation, Egger’s regression test did not detect evidence of small-study effects (*p* all > 0.10).

### Certainty of evidence

The certainty of evidence according to the GRADE framework is summarized in Table 4. The certainty of evidence was rated as low for daytime DBP variability and very low for the remaining outcomes. The lower certainty ratings were primarily attributable to the observational nature of the included studies and substantial unexplained heterogeneity for nighttime SBP variability, nighttime DBP variability, daytime SBP variability, 24-h SBP variability, and 24-h DBP variability.

**Table 4:** GRADE assessment of certainty of evidence.

Outcome	Studies (datasets)	Effect estimate (MD, 95% CI)	GRADE certainty	Reasons for rating
Nighttime SBP variability	11 (17)	1.89 (1.07–2.70)	Very low	Observational studies; downgraded for serious inconsistency (*I*^2^ = 85%)
Nighttime DBP variability	10 (16)	1.93 (1.17–2.69)	Very low	Observational studies; downgraded for serious inconsistency (*I*^2^ = 86%)
Daytime SBP variability	10 (16)	1.17 (0.35–1.99)	Very low	Observational studies; downgraded for serious inconsistency (*I*^2^ = 77%)
Daytime DBP variability	11 (17)	0.61 (0.26–0.96)	Low	Observational studies
24-h SBP variability	8 (12)	1.77 (0.80–2.75)	Very low	Observational studies; downgraded for serious inconsistency (*I*^2^ = 84%)
24-h DBP variability	9 (13)	1.17 (0.47–1.87)	Very low	Observational studies; downgraded for serious inconsistency (*I*^2^ = 85%)

GRADE assessment: Certainty of evidence started at low because all included studies were observational and predominantly cross-sectional. Outcomes with substantial unexplained heterogeneity (*I*^2^ > 75%) were downgraded one level for inconsistency. No further downgrading was applied for indirectness, imprecision, or publication bias because the study populations, exposure definitions, and outcome measurements were directly relevant to the review question, confidence intervals were reasonably precise, and Egger’s tests did not suggest significant small-study effects. CI, confidence interval; DBP, diastolic blood pressure; GRADE, Grading of Recommendations Assessment, Development and Evaluation; *I*^2^, inconsistency statistic; MD, mean difference; SBP, systolic blood pressure.

## Discussion

This meta-analysis provides a comprehensive synthesis of current evidence on the association between OSAS and BPV. The findings indicate that OSAS is consistently associated with increased BPV across different time periods, with the most pronounced differences observed during nighttime. Although all pooled analyses showed statistically significant associations, the 95% PIs crossed the null value for each outcome, suggesting that the magnitude of the association may vary across future study settings and populations, which is consistent with the substantial between-study heterogeneity observed in several analyses. Importantly, subgroup analyses suggest a potential gradient according to disease severity, particularly for nocturnal diastolic variability, supporting the hypothesis that the burden of sleep-disordered breathing may contribute to progressive dysregulation of blood pressure dynamics rather than merely elevating mean blood pressure levels.

The observed increase in BPV may also have important clinical implications because BPV has emerged as an independent predictor of cardiovascular morbidity and mortality. Previous studies have demonstrated that elevated BPV is associated with increased risks of stroke, coronary artery disease, heart failure, target-organ damage, and all-cause mortality, independent of mean blood pressure levels (35, 36). Therefore, the higher BPV observed among patients with OSAS may represent one potential pathway through which sleep-disordered breathing contributes to adverse cardiovascular outcomes. Although the present study did not evaluate cardiovascular events directly, the findings support the need for future longitudinal studies to determine whether BPV mediates the association between OSAS and cardiovascular risk.

Several pathophysiological mechanisms may explain the observed association between OSAS and increased BPV. A key pathway involves sympathetic nervous system activation, which is a hallmark of OSAS (37, 38). Recurrent episodes of hypoxia and arousal during sleep stimulate peripheral chemoreceptors and lead to sustained sympathetic overactivity, reflected by increased catecholamine levels and enhanced vasoconstrictor tone (39, 40). Experimental data indicate that sympathetic activity is closely related to BPV, particularly during nighttime, where apnea-related surges in blood pressure occur (41). In addition, repeated intrathoracic pressure swings and arousals may induce abrupt fluctuations in cardiac output and vascular resistance, contributing to short-term BP instability (42). These intermittent hemodynamic stresses may accumulate over time, leading to persistent alterations in vascular regulation. Endothelial dysfunction and arterial stiffness represent additional important mechanisms. Chronic intermittent hypoxia in OSAS promotes oxidative stress and systemic inflammation, impairing nitric oxide bioavailability and vascular reactivity (43, 44). These alterations reduce the buffering capacity of the arterial system and increase susceptibility to pressure fluctuations. Indeed, increased BPV has been linked to vascular damage, including arterial remodeling and target organ injury, independent of mean blood pressure levels (45). Moreover, disruption of normal circadian rhythms, including attenuation of nocturnal blood pressure dipping, is common in OSAS and may further amplify variability, particularly during sleep (19, 46). From a clinical perspective, these mechanisms suggest that BPV may serve as an integrative marker of the cumulative hemodynamic and neurohormonal burden imposed by OSAS.

The importance of controlling for age, sex, and BMI should be emphasized when interpreting the association between OSAS and BPV. These factors are strongly linked to both OSAS and cardiovascular regulation. For example, aging and obesity are associated with increased sympathetic activity (47) and arterial stiffness (48, 49), both of which contribute to higher BPV. Prior studies have demonstrated that the relationship between sympathetic activation and BPV persists even after adjustment for age and BMI, although these factors may still confound the magnitude of the association (41). In the present meta-analysis, all the included studies accounted for these key variables, enhancing the reliability of the observed association and reducing the likelihood that the findings are solely driven by baseline differences in patient characteristics.

The subgroup analyses provide additional insights into the potential mechanisms. The observation of a progressive increase in BPV with increasing OSAS severity, particularly during nighttime, is biologically plausible. Severe OSAS is characterized by more frequent and prolonged apneic events, leading to greater hypoxic burden, more pronounced sympathetic activation, and more severe disruption of sleep architecture. These factors may synergistically amplify short-term blood pressure fluctuations (50, 51). However, the lack of statistically significant differences across subgroups for some parameters suggests that the relationship may not be strictly linear or that the available data are limited and insufficient to detect modest differences. Variability in study design, sample size, and BPV assessment methods may also contribute to these findings. Sensitivity analyses further support the robustness of the results. The consistency of pooled estimates after sequential exclusion of individual datasets indicates that the association between OSAS and BPV is not driven by any single study. This is particularly important given the heterogeneity observed in some analyses. Such heterogeneity likely reflects differences in patient populations, ABPM protocols, clinical characteristics, and study methodologies. To further explore potential sources of heterogeneity, we performed meta-regression analyses using mean age, proportion of men, and mean BMI, which were among the most consistently reported study-level characteristics. However, none of these variables significantly influenced any of the BPV outcomes. These findings suggest that demographic differences alone are unlikely to explain the observed heterogeneity. Other factors, including differences in OSAS severity distribution, antihypertensive medication use, baseline blood pressure status, comorbidities, ABPM measurement protocols, and unmeasured clinical characteristics, may have contributed to the residual heterogeneity. Unfortunately, these factors could not be reliably evaluated because of inconsistent reporting across studies and the limited number of available datasets. In particular, almost all included studies enrolled hypertensive patients, with only one study including both hypertensive and non-hypertensive participants, precluding meaningful analyses according to hypertension status. Similarly, antihypertensive medication use was variably reported and could not be systematically evaluated.

This meta-analysis has several strengths. First, it is based on an up-to-date and comprehensive literature search, incorporating evidence from multiple databases, including both English and Chinese sources. It included 11 studies comprising 17 datasets and more than 2,000 participants, providing a relatively large evidence base for evaluating the association between OSAS and BPV. Second, the diagnosis of OSAS was consistently based on objective sleep assessments, predominantly polysomnography, ensuring accurate exposure classification. Third, BPV was assessed using standardized ABPM-derived measures, which represent the most widely accepted approach for evaluating short-term variability in clinical research. Finally, all the included studies considered key confounding factors such as age, sex, and BMI, improving the internal validity of the findings.

However, several limitations should be acknowledged. First, the majority of included studies were conducted in Asian populations, particularly in China, which may limit the generalizability of the findings to other populations. Second, all included studies were observational and largely cross-sectional, precluding causal inference. Although a plausible mechanistic link exists, the directionality of the association cannot be definitively established. Third, BPV was primarily assessed using standard deviation, while other metrics such as average real variability (ARV) and coefficient of variation were less frequently reported (52). Given that different BPV indices may capture distinct aspects of variability, the findings may not fully reflect the complexity of BPV. Fourth, substantial heterogeneity was observed in several analyses. Although additional meta-regression analyses showed that mean age, proportion of men, and mean BMI were not significant sources of heterogeneity, other potentially important factors, including OSAS severity, antihypertensive treatment, baseline blood pressure levels, ABPM protocols, and comorbidities, could not be comprehensively evaluated because of limited reporting and the study-level nature of the available data. Fifth, residual confounding cannot be excluded, particularly regarding antihypertensive treatment, sleep duration, and lifestyle factors. Although several studies adjusted for antihypertensive medication use, detailed information regarding specific medication classes, dosages, and treatment duration was not consistently reported. Therefore, the potential influence of individual antihypertensive agents on BPV could not be evaluated and remains an important topic for future investigation. Finally, publication bias cannot be entirely ruled out due to the limited datasets included for some outcomes, such as 24-h BPV, although formal testing did not suggest significant small-study effects.

From a clinical perspective, the findings of this study suggest that BPV may represent an important, yet under-recognized, pathway linking OSAS to cardiovascular risk. The observation that BPV is particularly elevated during nighttime is clinically relevant, as nocturnal blood pressure abnormalities have been strongly associated with adverse outcomes (53). These results support the potential value of incorporating ABPM and BPV assessment into the evaluation of patients with OSAS, especially those with moderate to severe disease. However, given the limitations of the available evidence, these implications should be interpreted with caution. Future research should aim to address the current gaps in evidence. Well-designed prospective cohort studies and randomized controlled trials are needed to clarify the causal relationship between OSAS and BPV and to determine whether interventions targeting OSAS, such as continuous positive airway pressure (CPAP), can effectively reduce BPV and improve cardiovascular outcomes. Standardization of BPV measurement and reporting, including the use of multiple indices, would enhance comparability across studies. In addition, studies incorporating individual participant data would allow for more detailed exploration of effect modifiers, including age, sex, BMI, comorbidities, and treatment factors.

## Conclusion

In conclusion, this meta-analysis suggests that OSAS is associated with increased BPV, particularly during nighttime, and a potential severity-dependent pattern may exist. These findings provide additional insight into the pathophysiological relationship between OSAS and cardiovascular dysfunction and support the hypothesis that impaired blood pressure regulation may represent an important mechanism linking OSAS to adverse cardiovascular outcomes. However, the certainty of evidence ranged from low to very low, and substantial heterogeneity was observed across several analyses. Therefore, the findings should be interpreted cautiously. Future well-designed prospective studies, particularly in non-Asian populations and more diverse clinical settings, are needed to confirm the reproducibility and generalizability of these findings, clarify potential effect modifiers, and determine their clinical relevance.

## Data Availability

The original contributions presented in the study are included in the article/Supplementary material, further inquiries can be directed to the corresponding author.
