# Supplementary material for: Association between obstructive sleep apnea syndrome and blood pressure variability: a meta-analysis
Source: Front Med (Lausanne). 2026 Jul 6;13:1882002. doi: 10.3389/fmed.2026.1882002 (PMC13381633; doi:10.3389/fmed.2026.1882002)
Supplement: Supplementary file 1 [file Supplementary_file_1.docx]

**Detailed search strategy for each database**

**PubMed**

#1 "Sleep Apnea, Obstructive"[Mesh] OR "obstructive sleep apnea"[tiab] OR OSA[tiab] OR OSAS[tiab] OR "sleep-disordered breathing"[tiab]

#2 "Blood Pressure Monitoring, Ambulatory"[Mesh] OR "ambulatory blood pressure monitoring"[tiab] OR ABPM[tiab] OR "24-hour blood pressure"[tiab] OR "24 hour blood pressure"[tiab] OR "daytime blood pressure"[tiab] OR "nighttime blood pressure"[tiab] OR "nocturnal blood pressure"[tiab]

#3 "Blood Pressure"[Mesh] AND ("variability"[tiab] OR "blood pressure variability"[tiab] OR BPV[tiab] OR "blood pressure variation"[tiab] OR "blood pressure fluctuation"[tiab] OR "short-term blood pressure variability"[tiab] OR "24-hour blood pressure variability"[tiab] OR "ambulatory blood pressure variability"[tiab] OR "standard deviation"[tiab])

#4 #1 AND #2 AND #3

**Embase**

#1 'obstructive sleep apnea'/exp OR 'sleep disordered breathing'/exp OR 'obstructive sleep apnea':ti,ab OR OSA:ti,ab OR OSAS:ti,ab

#2 'ambulatory blood pressure monitoring'/exp OR 'ambulatory blood pressure monitoring':ti,ab

OR ABPM:ti,ab OR '24 hour blood pressure':ti,ab OR 'daytime blood pressure':ti,ab OR 'nighttime blood pressure':ti,ab OR 'nocturnal blood pressure':ti,ab

#3 'blood pressure variability'/exp OR 'blood pressure variability':ti,ab OR BPV:ti,ab OR 'blood pressure variation':ti,ab OR 'blood pressure fluctuation':ti,ab OR 'short term blood pressure variability':ti,ab OR '24 hour blood pressure variability':ti,ab OR 'standard deviation':ti,ab

#4 #1 AND #2 AND #3

**Web of Science**

TS=(("obstructive sleep apnea" OR OSA OR OSAS OR "sleep-disordered breathing") AND("blood pressure variability" OR "BP variability" OR BPV OR "blood pressure variation" OR "blood pressure fluctuation" OR "short-term blood pressure variability" OR "24-hour blood pressure variability" OR "ambulatory blood pressure variability" OR "standard deviation")AND ("ABPM" OR "ambulatory blood pressure monitoring" OR "24-hour blood pressure" OR "24 hour blood pressure" OR "daytime blood pressure" OR "nighttime blood pressure" OR "nocturnal blood pressure"))

**Wanfang**

主题 = (("阻塞性睡眠呼吸暂停" OR "睡眠呼吸暂停综合征" OR "OSA" OR "OSAS" OR "睡眠呼吸障碍") AND ("血压变异性" OR "血压波动" OR "血压变异" OR "血压变动" OR "血压标准差") AND ("动态血压监测" OR "24小时血压" OR "24 h血压" OR "昼间血压" OR "夜间血压" OR "夜间血压"))

**CNKI**

主题 = (("阻塞性睡眠呼吸暂停" OR "睡眠呼吸暂停综合征" OR "OSA" OR "OSAS" OR "睡眠呼吸障碍") AND ("血压变异性" OR "血压波动" OR "血压变异" OR "血压变动" OR "血压标准差") AND ("动态血压监测" OR "24小时血压" OR "昼间血压" OR "夜间血压" OR "夜间血压"))
