# Supplementary material for: Association between obstructive sleep apnea syndrome and blood pressure variability: a meta-analysis
Source: Front Med (Lausanne). 2026 Jul 6;13:1882002. doi: 10.3389/fmed.2026.1882002 (PMC13381633; doi:10.3389/fmed.2026.1882002)

**Supplemental Figure 1** Forest plots of subgroup analyses according to severity of OSAS on nighttime BPV as evaluated by SD of nighttime SBP and DBP. (A) Subgroup analysis according to the severity of OSAS on SD of nighttime SBP; and (B) Subgroup analysis according to the severity of OSAS on SD of nighttime DBP;


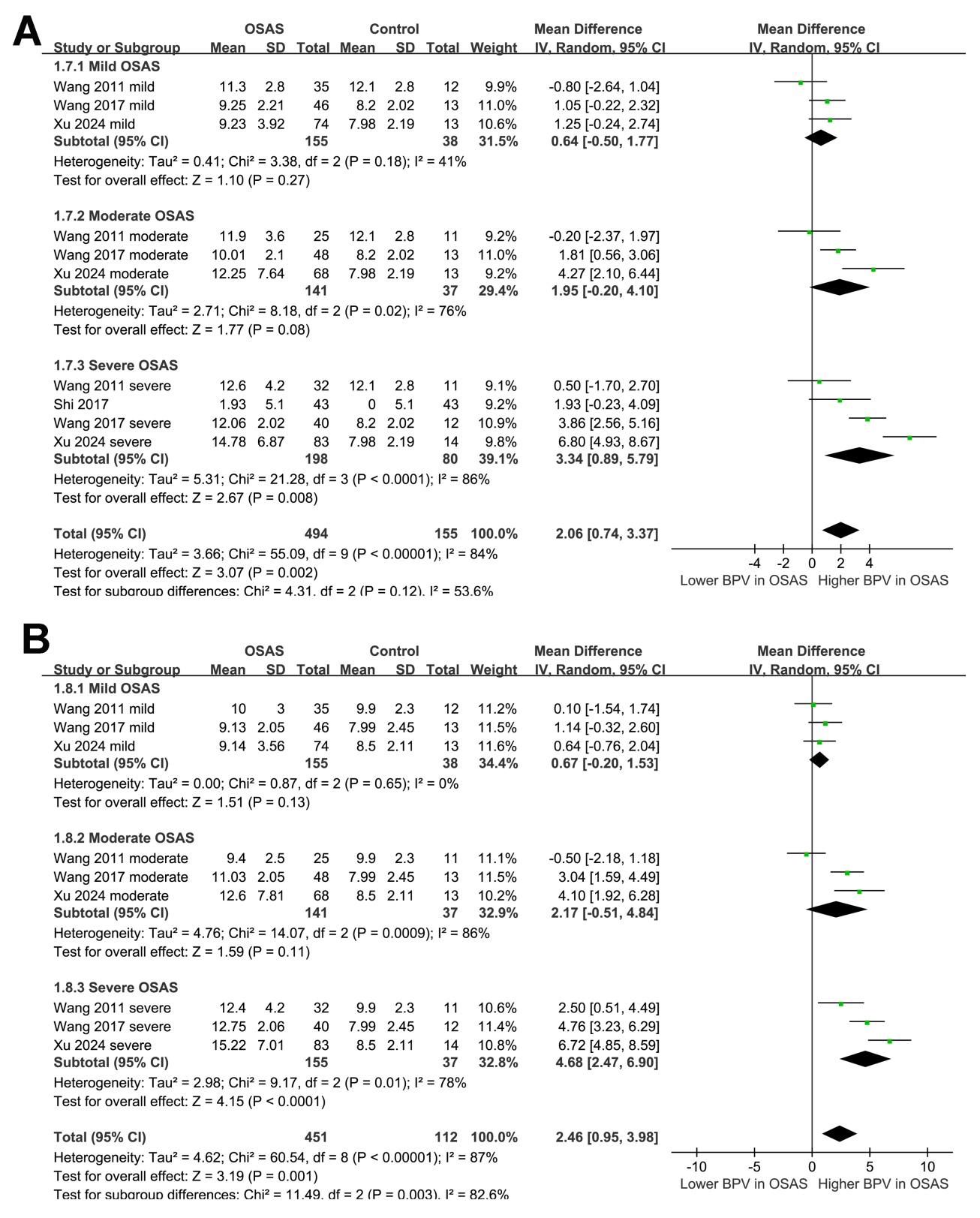

Supplement: Supplementary file 2 [file Table_1.DOCX]
