# Supplementary material for: Association between obstructive sleep apnea syndrome and blood pressure variability: a meta-analysis
Source: Front Med (Lausanne). 2026 Jul 6;13:1882002. doi: 10.3389/fmed.2026.1882002 (PMC13381633; doi:10.3389/fmed.2026.1882002)

**Supplemental Figure 2** Forest plots of subgroup analyses according to severity of OSAS on daytime BPV as evaluated by SD of nighttime SBP and DBP. (A) Subgroup analysis according to the severity of OSAS on SD of daytime SBP; and (B) Subgroup analysis according to the severity of OSAS on SD of daytime DBP;


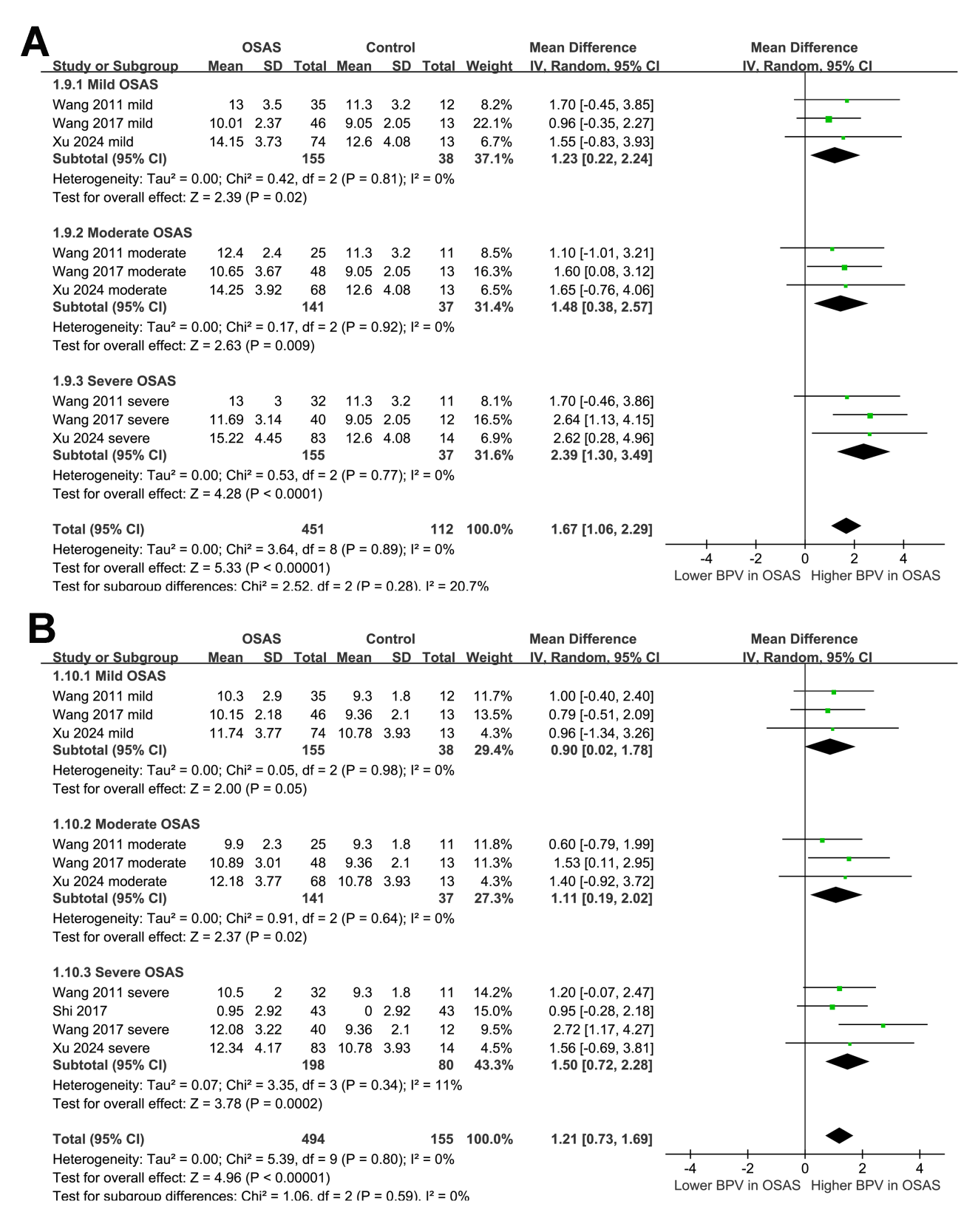

Supplement: Supplementary file 3 [file Table_2.DOCX]
