# Supplementary material for: Association between obstructive sleep apnea syndrome and blood pressure variability: a meta-analysis
Source: Front Med (Lausanne). 2026 Jul 6;13:1882002. doi: 10.3389/fmed.2026.1882002 (PMC13381633; doi:10.3389/fmed.2026.1882002)

**Supplemental Figure 3** Forest plots of subgroup analyses according to severity of OSAS on daytime BPV as evaluated by SD of 24-hour SBP and DBP. (A) Subgroup analysis according to the severity of OSAS on SD of 24-hour SBP; and (B) Subgroup analysis according to the severity of OSAS on SD of 24-hour DBP;


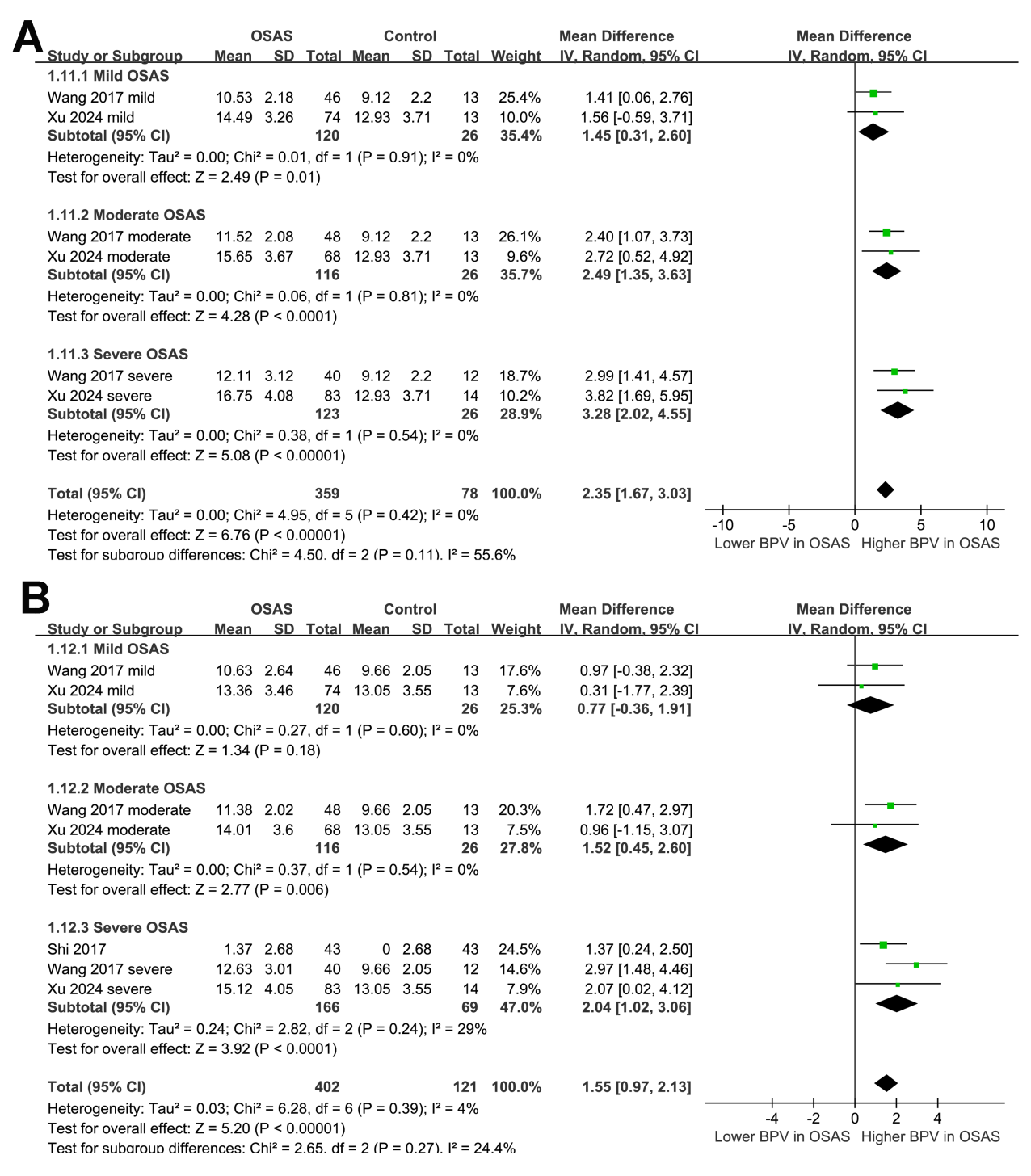

Supplement: Supplementary file 4 [file Table_3.DOCX]
